# Analysis of diet-related stroke disease burden in China from 1990 to 2021 and projections for 2025–2044

**DOI:** 10.3389/fnut.2025.1571916

**Published:** 2025-06-02

**Authors:** Zhe Wang, Wenjuan Pei, Meili Gou, Chen Gao

**Affiliations:** Department of General Practice, The 940th Hospital of Joint Logistics Support Force of Chinese People's Liberation Army, Lanzhou, China

**Keywords:** stroke, diet, disease burden, prediction, China

## Abstract

**Objective:**

To analyze the burden of diet-related stroke diseases in China from 1990 to 2021 and to predict its trends in the next 20 years.

**Method:**

Extract data from the 2021 Global Burden of Disease Database and analyze the burden of diet-related stroke d isease in China from 1990 to 2021. And using a time series model to predict the burden of diet related stroke disease in the next 20 years.

**Results:**

In 2021, the crude mortality rate of diet related stroke in China was 30.70/100,000, and the crude Disability Adjusted of Life Years (DALY) rate was 729.10/100,000. The standardized mortality rate of 21.59/100,000 and the standardized DALY rate of 485.83/100,000 in 2021 have decreased by 54.48 and 55.05%, respectively compared to 1990, but are higher than the levels of high Socio-demographic Index (SDI) regions worldwide. In 2021, the male attributable mortality rate and DALY rate were higher than those of females (39.39/100,000 vs. 21.59/100,000, 942.76/100,000 vs. 505.15/100,000), and the male and female attributable disease burden indicators showed a downward trend over time. In 2021, the mortality rate and DALY rate of diet related stroke were positively correlated with age. Compared to other types of stroke, ischemic stroke has the highest disease burden, and high salt still ranks first among various unhealthy dietary risk factors. Autoregressive Integrated Moving Average (ARIMA) model predicts that the burden of diet related stroke disease will significantly decrease in the next 20 years.

**Conclusion:**

From 1990 to 2021, the burden of diet-related stroke diseases in China has shown a downward trend, and the disease burden problem is more prominent in the older adult population and men. A series of effective measures should be taken to strengthen the healthy diet of key populations. In the next 20 years, the burden of diet-related stroke diseases will significantly decrease.

## 1 Introduction

The Global Burden of Disease study (GBD) shows that in 2019, the number of stroke patients in China was about more than 3 million, and the number of deaths was about more than 2 million (1). As a major global health issue, stroke is characterized by high mortality and disability rates, imposing a serious economic burden on families and society. The occurrence of stroke is closely related to multiple risk factors (2), among which dietary factors, as a controllable and important part, cannot be ignored. Therefore, analyzing the disease burden of diet-related stroke has important theoretical guiding significance for formulating effective prevention strategies and reducing the social and family burden it causes. However, research on the disease burden of diet-related stroke, especially exploring the relationship between the two from different time, age, and gender perspectives, is still lacking. Therefore, based on the GBD 2021 database, this study analyzed the disease burden of str oke attributed to poor diet in China from 1990 to 2021 using indicators such as mortality rate and Disability Adjusted Life Years (DALY), and predicted the disease burden of diet-related stroke in the next 20 years. This study deepens citizens' understanding of the occurrence of diseases caused by poor eating habits by evaluating the impact of poor diet on the disease burden of stroke, and improves the scientificity and effectiveness of formulating relevant strategies. This has important public health significance for protecting public health, reducing the fatality rate of stroke, alleviating the social economic burden, and improving people's health level and quality of life. The research results provide a scientific basis and guiding suggestions for formulating scientific diet control strategies, and can further promote the control of poor diet.

## 2 Materials and methods

### 2.1 Data source

The research data mainly come from the GBD 2021 database. This database has evaluated the disease burden caused by 369 diseases and 87 risk factors in 204 countries (3–5). The data on diet-related stroke evaluated in the GBD 2021 database mainly come from the death cause monitoring data of the Chinese Center for Disease Control and Prevention's Chronic Noncommunicable Disease Control Center, etc. (6–8).

### 2.2 Assessment method of attributable disease burden

In the GBD 2021 database, the theoretical minimum risk exposure level (TMREL) is quantified for each risk factor (9–11). The proportion of the disease burden of stroke caused by poor diet in the target population to the overall disease burden, that is, the population attributable fraction (PAF), and the estimated value of the disease burden of stroke attributed to poor diet is the indicator of a given age, gender, location, and year multiplied by PAF (12–15).

### 2.3 Statistical method

Referring to the data of the disease burden of stroke caused by poor diet from 1990 to 2021, the time series model method was used for modeling. The time series model predicts future trends. By selecting an appropriate model, performing parameter estimation and model validation based on historical data, a model that can accurately predict future values is constructed (16). After identifying the attributes of the model, the Autoregressive Integrated Moving Average (ARIMA) model was selected (17). The Augmented Dickey-Fuller (ADF) test method was used to determine whether the time series is stationary. If the data is not stationary, differencing is required. According to the ADF test results, the non-stationary time series is differenced until the series is stationary. The number of differences *d* is the *d* parameter in the ARIMA model. Analyze the autocorrelation coefficient of the time series and observe whether it is trailing or truncated to determine the number of autoregressive terms *p*. Analyze the partial autocorrelation coefficient of the time series, and also observe whether it is trailing or truncated to determine the number of moving average terms *q*, and determine the optimal model for modeling (18). The white noise test was used to evaluate the model, and the disease burden of stroke caused by poor diet in the next 20 years was predicted. The test level α = 0.05 (19).

## 3 Results

### 3.1 The disease burden of stroke attributed to dietary risk factors in China and its changing trends

In 2021, the crude mortality rate of stroke attributed to poor diet in China was 30.70 per 100,000, and the crude DALY rate was 729.10 per 100,000. The disease burden was the highest in the age group of ≥70 years old, and the crude mortality rate and crude DALY rate of males were significantly higher than those of females. From 1990 to 2021, the changing trends of the mortality rate and DALY rate of stroke attributed to poor diet were slightly different. Taking 2000 and 2010 as nodes, the attributable mortality rate first increased slowly, then decreased slowly, and finally increased slowly again; the attributable DALY rate showed a trend of continuous decrease followed by a slow increase. In 2021, the mortality rate increased by 4.62% compared to 1990, and the DALY decreased by 6.14% (Table 1). Analyzing the changing trends of the attributable disease burden of different genders, it was found that the attributable mortality rate and DALY rate of males generally showed a slow and stable upward trend. From 1990 to 2021, the increase ranges were 19.46 and 6.01%, respectively, while the attributable mortality rate and DALY rate of females both showed a downward trend, with the decrease ranges being 15.25 and 23.12%, respectively (Table 1).

**Table 1:** Diet-related stroke disease burden in China, 1990–2021.

**Characteristics**	**Mortality rate (/100,000)**			**DALY rate (/100,000)**		
	**Male**	**Female**	**Total**	**Male**	**Female**	**Total**
**Age group 15–49 years**						
1990	4.85	2.92	3.92	254.74	168.68	213.16
2000	5.03	2.58	3.84	261.65	151.03	207.81
2010	4.40	1.67	3.06	228.28	103.54	167.00
2021	3.33	1.08	2.25	180.24	77.49	130.88
Percentage change	−31.35	−63.01	−42.60	−29.24	−54.06	−38.60
**Age group 50–69 years**						
1990	124.79	85.68	105.93	3,847.38	2,689.55	3,289.16
2000	103.75	61.77	83.45	3,183.83	1,951.34	2,587.75
2010	75.40	35.94	55.99	2,376.69	1,193.12	1,794.42
2021	57.20	25.49	41.36	1,833.40	879.78	1,356.93
Percentage change	−54.16	−70.25	−60.96	−52.35	−67.29	−58.75
**Age group** ≥**70 years**						
1990	515.78	340.14	416.42	8,933.41	5,680.55	7,093.35
2000	463.13	280.32	362.96	7,861.39	4,557.54	6,050.97
2010	388.53	208.03	291.57	6,450.66	3,307.16	4,761.99
2021	305.16	151.43	221.71	5,046.37	2,454.30	3,639.37
Percentage change	−40.84	−55.48	−46.76	−43.51	−56.80	−48.69
**Total population**						
1990	32.98	25.47	29.34	889.27	657.02	776.82
2000	35.73	24.20	30.14	920.10	597.27	763.59
2010	37.23	21.06	29.33	926.76	502.20	719.20
2021	39.39	21.59	30.70	942.76	505.15	729.10
Percentage change	19.46	−15.25	4.62	6.01	−23.12	−6.14

Analyzing the attributable disease burden of each age group, it was found that from 1990 to 2021, the attributable mortality rate and DALY rate of all age groups showed a downward trend. Among them, the age group of 50–69 years old had the largest decrease range, which were 60.96 and 58.75%, respectively. In addition, the disease burden of the age group of ≥70 years old accounted for the highest proportion among all age groups (Table 1).

**Figure 1:**
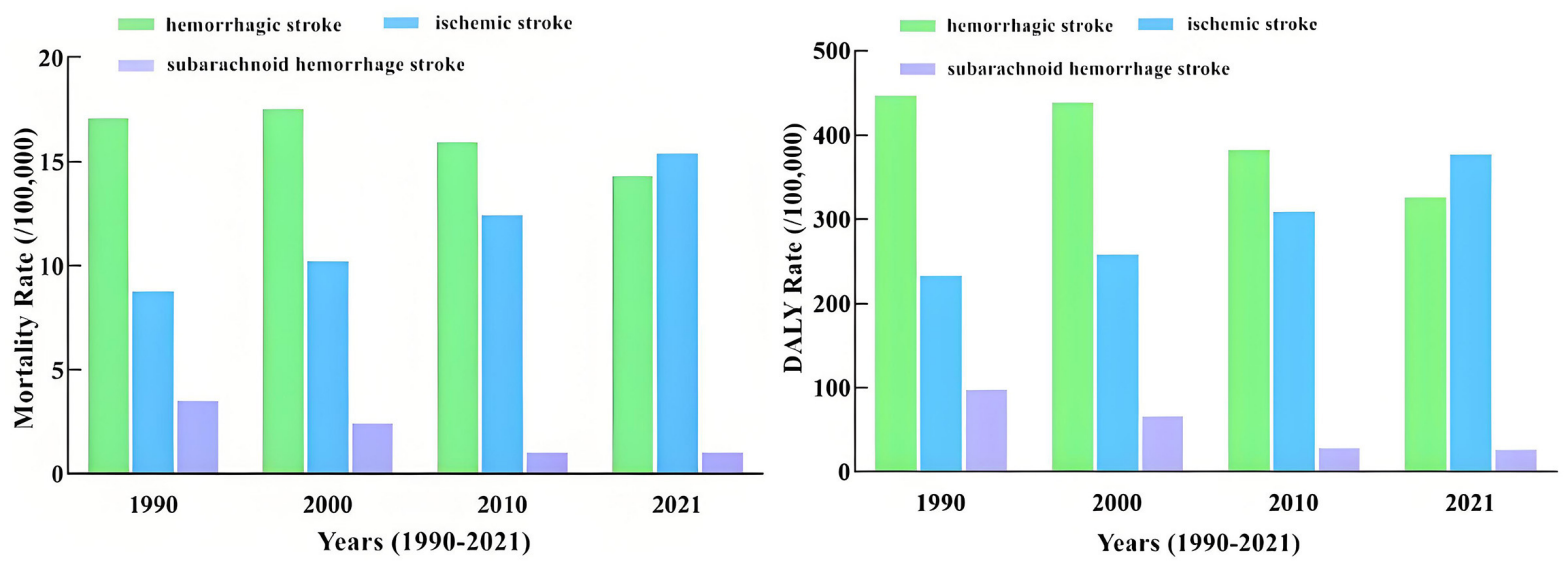
Disease burden of stroke by type in China, 1990–2021.

Analyzing the disease burden of three types of stroke, it was found that before 2021, the type of stroke with the highest diet-related disease burden was hemorrhagic stroke, followed by ischemic stroke, and subarachnoid hemorrhage stroke ranked third. However, over time, the gap between hemorrhagic and ischemic stroke continued to narrow. In 2021, the disease burden of diet-related ischemic stroke surpassed that of hemorrhagic stroke and rose to the first place (Figure 1).

### 3.2 The changing situations of the disease burden of diet-related stroke in China and different socio-demographic index (SDI) regions worldwide

**Figure 2:**
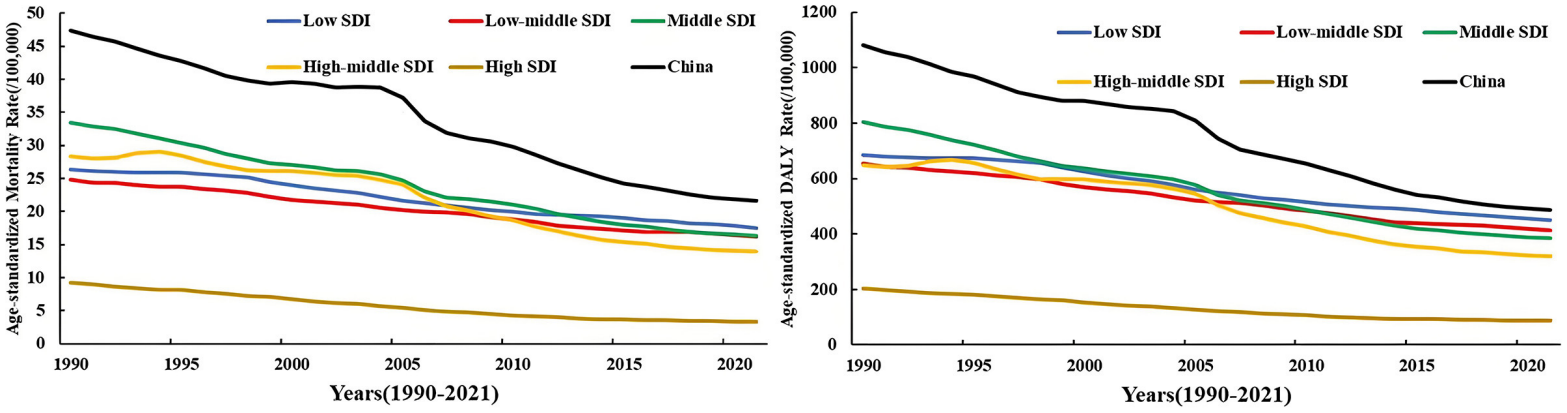
Disease burden of stroke in China and the world with different sociodemographic indices, 1990–2021.

From 1990 to 2021, the age-standardized mortality rate and DALY rate of stroke attributed to dietary risk factors in China generally showed a downward trend. The standardized mortality rate decreased from 47.44 per 100,000 in 1990 to 21.59 per 100,000 in 2021, with a change rate of −54.48%, which was close to the change rate of high SDI regions (−63.94%); in 1990, the standardized DALY rate was 1,080.82 per 100,000, and in 2021, it was 485.83 per 100,000, a decrease of 55.05%, second only to the decrease rate of high SDI regions (57.4%). From 1990 to 2021, the disease burden of diet-related stroke in China was higher than that of different SDI regions worldwide. However, over time, the gap between the attributable disease burden in China and that in high SDI regions worldwide became smaller and smaller (Figure 2).

### 3.3 Ranking and changes of mortality rate and DALY rate of stroke attributable to unhealthy dietary risk factors in 1990 and 2021

**Figure 3:**
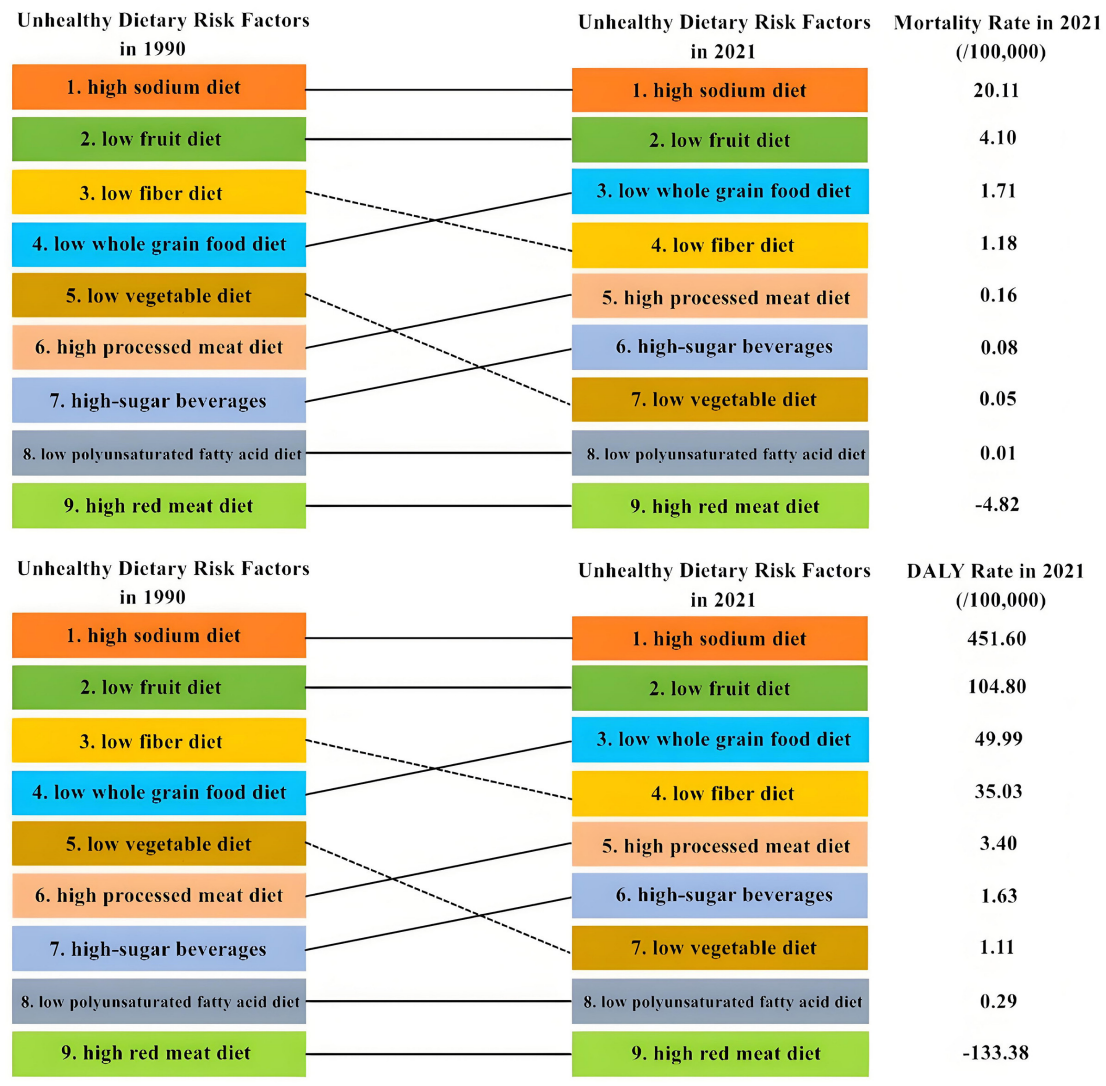
Sequential changes in stroke burden of disease attributable to poor dietary risk factors in China in 1990 and 2021.

Among the nine unhealthy dietary risk factors, in 2021, the age-standardized mortality rate and DALY rate of stroke attributable to high-sodium diet decreased significantly. They dropped from 39.04 per 100,000 and 875.5 per 100,000 in 1990 to 20.11 per 100,000 and 451.6 per 100,000 in 2021 respectively. However, high sodium still ranked first in the disease burden of diet-related stroke. In 1990 and 2021, low fruit diet and low whole grain food diet ranked second and third respectively in the disease burden of diet-related stroke. The disease burden of stroke related to low fiber diet and low vegetable diet decreased significantly. Their rankings dropped from the 3rd and 5th place in 1990 to the 4th and 7th place in 2021, respectively. However, the rankings of the mortality rate and DALY rate of stroke related to high-sugar beverages, high processed meat diet, and low whole grain food all increased (Figure 3).

### 3.4 Model construction and prediction

**Figure 4:**
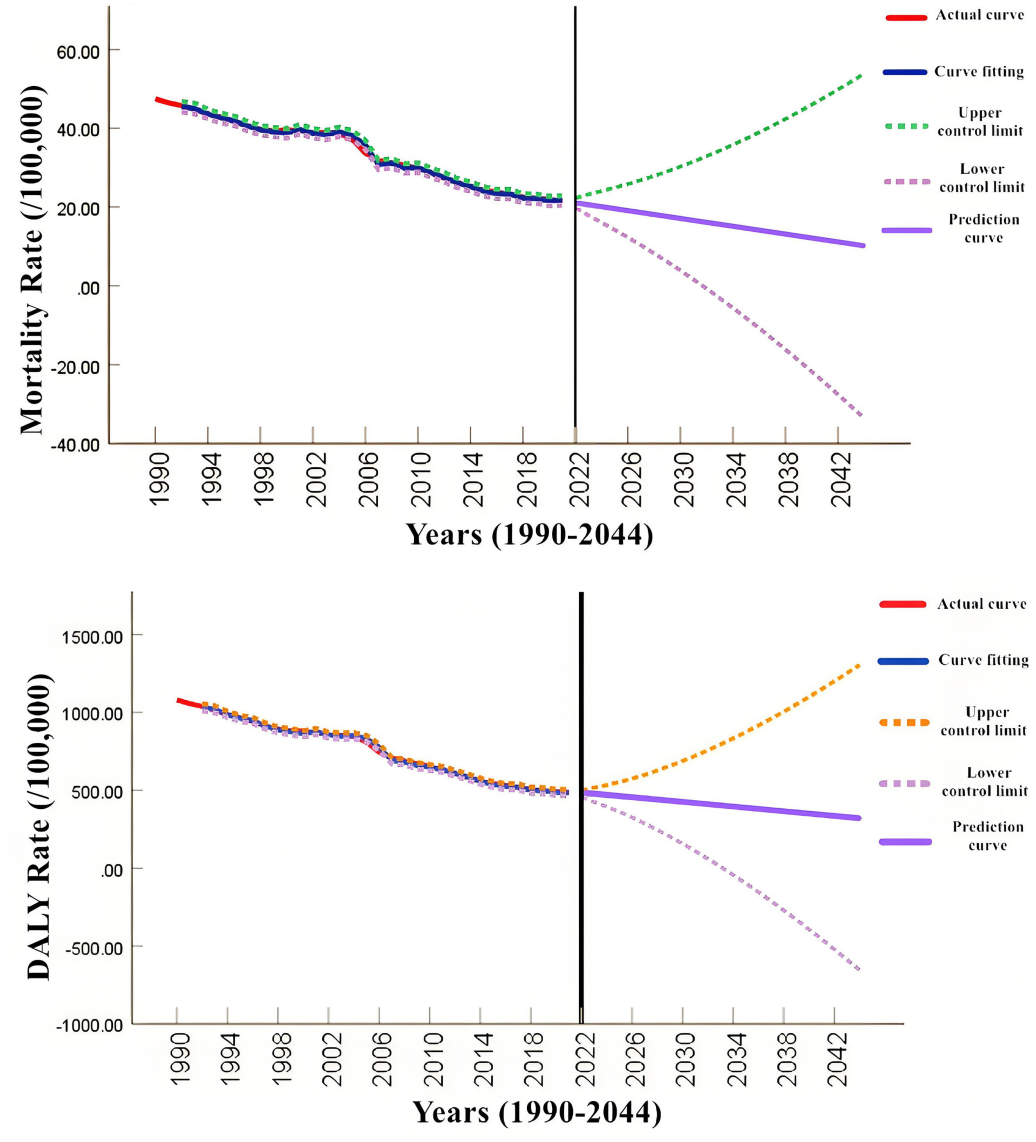
Prediction of diet-related stroke burden in China, 2022–2044.

Based on the age-standardized mortality rate and DALY rate of diet-related stroke from 1990 to 2021 as the original data, the disease burden of stroke in China attributable to unhealthy dietary risk factors from 2022 to 2044 was predicted. Using Statistical Analysis System (SAS) software and through the Akaike Information Criterion (AIC) and Schwarz Bayesian Criterion (SBC) index tests, the optimal model for the standardized mortality rate was determined to be ARIMA (0, 2, 2), with Coefficient of Determination *R*^2^ of 0.995. The optimal model for the standardized DALY rate was ARIMA (2, 2, 0), with Coefficient of Determination *R*^2^ of 0.997. To ensure the robustness and accuracy of the prediction model, the data set was divided into the training set (1990–2015) and the test set (2016–2021). The training set is used for model training and parameter optimization, and the test set is used to evaluate the generalization ability of the model. The prediction results on the test set: MSE is 25, RMSE is 5, MAE is 5, *R*^2^ is 0.98, which has good robustness and the effectiveness of predicting the future disease burden. The model prediction results show that from 2022 to 2044, both the standardized mortality rate and DALY rate of diet-related stroke in China will show a downward trend. The standardized mortality rate will decrease from 19.3 per 100,000 in 2022 to 10.12 per 100,000 in 2044. The standardized DALY rate will decrease from 457.38 per 100,000 in 2022 to 325.44 per 100,000 in 2044 (Figure 4).

## 4 Discussion

The results of GBD 2019 show that stroke has become the second leading cause of death globally. The risk factors for stroke are diverse, including modifiable and non-modifiable factors (20). Studies have found that high salt, high sugar, low fruit intake, etc. are closely related to the occurrence of stroke (21). Therefore, it is self-evident that studying the impact of controllable dietary factors on the disease burden of stroke is important. Based on the GBD 2021 database, this study deeply analyzed the evolution of the disease burden of stroke in China caused by dietary factors from 1990 to 2021 and further predicted the possible development trend of this burden in the next 20 years. The results show that from 1990 to 2021, the crude incidence rate and DALY rate of diet-related stroke showed an upward trend, while the attributable standardized incidence rate and standardized DALY rate showed a significant downward trend. It is predicted that they will continue to decline in the next 20 years. The increase in the crude incidence rate and DALY rate may be due to the intensification of population aging in China, leading to an increase in the size of the older adult population. In addition, the older adult are a high-risk group for stroke. Therefore, the crude incidence rate and DALY rate of stroke still show an upward trend. The downward trends of the standardized rates and the predicted disease burden of diet-related stroke in the next 20 years indicate that with the rapid economic development in China over the past few decades, the dietary structure of Chinese residents has gradually improved, reducing the burden of diet-related stroke. The above trends are consistent with the findings of Kim et al.'s (22) study on diet-related stroke in East Asia.

The disease burden of diet-related stroke in China is significantly higher in men than in women. Compared with men, the disease burden of stroke caused by unhealthy eating habits in women has a greater decline. The reasons may be that men generally have higher blood pressure and cholesterol levels than women, increasing the risk of stroke (23). On the other hand, compared with women, men may be more inclined to consume high-fat, high-salt and other foods. These unhealthy eating habits are important risk factors for stroke (24–26). In addition, the protective effects of estrogen on women include dilating arteries, regulating lipid metabolism, etc. Therefore, under the same poor dietary conditions, women may relatively reduce the occurrence of stroke due to the protective effect of estrogen (27).

In terms of age, Friedman et al. (28) found that the older adult are more susceptible to stroke caused by poor diet due to physical decline, and have poor recovery ability, resulting in increased disease burden. The disease burden of diet-related stroke in China increases with age. The disease burden of stroke is the highest in the age group of ≥70 years old, and the 50–69 age group has the largest decline in attributable disease burden. The reasons for this phenomenon may be that physiological changes caused by aging and the cumulative effects of long-term unhealthy eating habits lead to the concentration of disease burden mainly in the age group of ≥70 years old. The significant decline in the attributable disease burden of the 50–69 age group may be related to factors such as changes in the lifestyle of this age group, the popularization of health education, and the effectiveness of policy interventions. The disease burden of diet-related stroke is mainly reflected in two types of diseases: ischemic stroke and hemorrhagic stroke. In recent years, the disease burden of ischemic stroke has exceeded that of hemorrhagic stroke. The reason may be that people's dietary structure has changed significantly in recent years (29–31). In terms of environmental factors, Chen et al. (32) have shown that air pollution and poor diet work together to significantly increase the risk of stroke and disease burden. In terms of genetic factors, Yang et al. (33) found that through genome-wide association studies, specific genetic variants affect the body's metabolism of harmful substances in the diet, which in turn affects the incidence of stroke. The intake of high-fat, high-salt, and high-sugar foods has increased. These unhealthy eating habits are important factors leading to atherosclerosis and thrombosis, thus greatly increasing the risk of ischemic stroke.

Olesen et al. (34) compared different countries in Europe and found that the burden of stroke disease in high SDI countries decreased significantly through dietary intervention. Compared with different global SDI regions, the disease burden of diet-related stroke in China has always been at a relatively high level. However, the gap between the disease burden of stroke in China and that in high SDI regions is getting smaller and smaller. In 2021, the disease burden of stroke closely related to low vegetable and low fiber eating habits and its ranking among stroke risk factors both experienced a significant decline. Studies have found that long-term adherence to a healthy lifestyle, increasing the intake of fruits and vegetables in the diet, and limiting red meat intake can reduce the risk of ischemic stroke by 39% (35). In recent years, the dietary structure of Chinese residents has changed significantly. The intake of foods such as vegetables and fruits has gradually increased, and the dietary structure has become more diverse and balanced. On the other hand, the government and all sectors of society have increased the publicity of healthy eating, popularized the knowledge and concepts of healthy eating, and improved the public's awareness of the harm of unhealthy eating habits. The rapid economic development also plays an important role. However, compared with high SDI regions, the disease burden of stroke attributable to unhealthy diet in China is still relatively high. Therefore, it is necessary to further strengthen the government's prevention and control strategies and improve the public's awareness to narrow the gap with high SDI regions. In the next 20 years, it is expected that the disease burden of stroke induced by unhealthy eating habits will show a gradually decreasing trend.

From a global perspective, prevention and control measures and effects vary in different regions. In Africa, Adebayo et al. (36) improved the diet of residents through community health education programs, which effectively reduced the incidence of stroke. In North America, Smith et al. (37) found that although the overall diet health awareness of residents is high, some people still face a higher risk of stroke due to economic and cultural factors. In terms of disease burden prediction, in addition to time sequence models, machine learning algorithms are widely used. Liu et al. (38) use deep learning models combined with multi-source data to accurately predict the trend of stroke disease burden. In the study of the mechanism of diet and stroke, Wang et al. (39) found that intestinal microbiota metabolites play a key role in the process of stroke caused by poor diet, providing new targets for prevention and treatment.

In conclusion, from 1990 to 2021, although the disease burden of stroke closely related to dietary factors in China showed a downward trend, its level was still significantly higher than that in high SDI regions globally. Among them, ischemic stroke, as one of the types of stroke induced by unhealthy eating habits, has a particularly significant impact. It is worth noting that men and the older adult are more vulnerable to diet-related stroke risks and have become high-risk groups that require key attention. To effectively address this challenge, promoting a dietary pattern that is low in salt and fat, reduces the intake of processed meat, and increases the intake of fruits, vegetables, and dietary fiber is regarded as one of the key strategies for preventing stroke (40). In this process, the government plays a crucial role. By strengthening public health publicity and education, the government can deepen the public's understanding of the importance of healthy eating and stimulate the enthusiasm of all sectors of society to improve the dietary structure. At the same time, the public should also take the initiative to control unhealthy eating habits and actively participate in various public health activities to be responsible for the health of themselves and their families. For people already in a high-risk state, regular stroke screening and timely treatment are particularly important, which helps to detect and intervene in potential health risks at an early stage. Overall, in China, through the scientific guidance of government policies and the active efforts of the public, the disease burden of stroke caused by unhealthy diet is gradually decreasing. However, we still need to remain vigilant and take more scientific and comprehensive measures to further reduce the negative impact of unhealthy diet on the incidence of stroke and continuously improve the overall health level of the nation.

Limitations of this study: (i) Due to limitations in data resources, we were unable to conduct a detailed analysis of the disease burden of stroke closely related to diet in each province of China. This gap limits the geographical subdivision and in-depth understanding of the research conclusions. (ii) Although the study covers data from a long time period, the latest data have not been updated in the database in a timely manner, which may lead to the research results not fully reflecting the current situation. (iii) The prediction model assumes that the relationship between future eating habits and stroke risk remains unchanged. In reality, however, these relationships may be affected by various factors and change. Therefore, the prediction results may not fully conform to the actual data. (iv) The study included the distribution of risk factors and did not use a time series regression model to analyze the specific impact of these risk factors on the burden of stroke disease. However, considering the issues of data integrity and consistency, as well as the overfitting risks that model complexity may bring, the more robust ARIMA model was chosen. The ARIMA model was chosen for its maturity and efficiency in handling univariate time series data, but this might limit the in-depth understanding of the impact of risk factors. Time series regression models may provide additional insights, but they require more data and computing resources. (v) The ARIMA model was chosen for its maturity and efficiency in handling univariate time series data, but this might limit the in-depth understanding of the impact of risk factors. Time series regression models may provide additional insights, but they require more data and computing resources.

## Data Availability

The raw data supporting the conclusions of this article will be made available by the authors, without undue reservation.
